# Review on Lipase-Catalyzed Flavor Synthesis: Global Trends and Advances

**DOI:** 10.1021/acs.jafc.5c00386

**Published:** 2025-07-01

**Authors:** Artur Ramon Tomé Oliveira, Francisco Izaias da Silva Aires, Dayana Nascimento Dari, José Roberto de Matos Filho, Kaiany Moreira dos Santos, Francisco Simão Neto, Francisco Lucas de Souza Magalhães, Patrick da Silva Sousa, Paulo Gonçalves de Sousa Junior, Rafael Leandro Fernandes Melo, Iris Cornet, Jesús Fernández-Lucas, Marcos Carlos de Mattos, José Cleiton Sousa dos Santos

**Affiliations:** † Department of Organic and Inorganic Chemistry, Science Center, 28121 Federal University of Ceará, Campus do Pici, Fortaleza 60455-970, CE, Brazil; ‡ Institute of Engineering and Sustainable Development, 245069 University of International Integration of Afro-Brazilian Lusofonia (Unilab), Campus das Auroras, Redenção 62790-970, CE, Brazil; § Department of Chemical Engineering, UFC, Campus do Pici, Block 709, Fortaleza 60440-554, CE, Brazil; ∥ Department of Analytical Chemistry and Physical Chemistry, Science Center, UFC, Campus Pici, Fortaleza 60455-760, CE, Brazil; ⊥ Department of Metallurgical Engineering, Federal University of Ceara, Fortaleza 60440-554, CE, Brazil; # Campus Groenenborger, Groenenborgerlaan 171, Antwerpen 2020, Belgium; ∇ Applied Biotechnology Group, Universidad Europea de Madrid, Urbanización El Bosque, Villaviciosa de Odón 28670, Madrid, Spain; ○ Grupo de Investigación en Ciencias Naturales y Exactas, GICNEX, Universidad de la Costa, CUC, Calle 58 # 55-66, Barranquilla 080002, Colombia; ◆ Department of Biochemistry and Molecular Biology, Faculty of Biology, 16734 Universidad Complutense de Madrid, C. de José Antonio Novais, 12, Madrid 28040, Spain; ¶ Institute of Engineering and Sustainable Development, University of International Integration of Afro-Brazilian Lusofonia, Campus das Auroras, Redenção 62790-970, CE, Brazil

**Keywords:** esterified flavors, lipase biocatalysis, flavor ester synthesis trends

## Abstract

The enzymatic synthesis of esterified flavors is pivotal in the food, pharmaceutical, and cosmetic industries. Among the available approaches, lipase-catalyzed reactions have gained increasing attention due to the enzymes’ biodegradability, high enantio- and regioselectivity, availability, and effectiveness under mild, eco-friendly conditions, aligning well with the principles of green chemistry. This topic was selected due to the vast potential of biocatalysis in the sustainable and efficient synthesis of diverse flavor compounds. Using the Web of Science database with the keywords “flavor”, “enzyme”, and “lipase”, 189 relevant articles were identified, revealing that the majority of publications fall within the fields of chemistry, food science, and biochemistry. This review explores the catalytic mechanisms of lipases during acetylation reactions, focusing on structural conformational changes during enzymatic processes. Lipases from (notably CALB, Lipozyme, and Novozym 435) emerge as the most commonly employed biocatalysts for esterified flavor production. Interestingly, most studies emphasize the immobilization supports rather than the intrinsic properties or engineering of the enzymes themselves. The findings underscore a growing global interest in enzyme-mediated flavor synthesis. Future studies should explore enzyme engineering to enhance activity and specificity, broaden the range of usable substrates, and improve the operational stability. Key challenges include the high cost of enzyme production, limited substrate scope in some systems, and the need for scalable, cost-effective bioprocesses for industrial applications.

## Graphical abstract

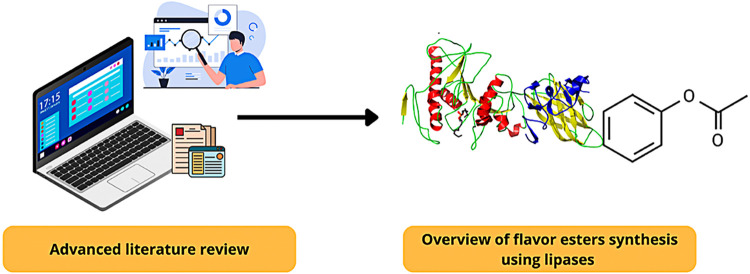

## 1 Introduction

One of the fundamental principles of green chemistry is based on studies showing that processes can increase reaction conversions while reducing the production of byproducts. Biocatalysis is an effective alternative to achieve these results.^1,2^ Literature indicates that using enzymes as biocatalysts is optimal due to their high specificity—chemo-, regio-, and enantioselectivity—and ability to function under mild reaction conditions such as moderate temperature, pressure, and pH.^3–8^

They are used in organic reactions to produce pharmaceuticals, biofuels, biolubricants, and cosmetics and have applications in the pharmaceutical industry.^9–12^ Literature reports highlight the versatility of lipases as biocatalysts in various reactions, including asymmetric esterification and transesterification, aminolysis, asymmetric hydrolysis, acidolysis, alcoholysis, etc.^13^

Esterified flavors are known for their versatility, making them essential in producing beverages, cosmetics, pharmaceuticals, and foods due to their flavor and fragrance properties.^14^ The synthesis of an ester flavor involves an esterification reaction between an alcohol, which acts as a nucleophile, and an organic acid, which acts as an electrophile. Flavor and aroma from these esters are directly related to the chemical nature of the reagents used in the synthesis.^15^

The industry has been exploring new biotechnological methods for flavor ester synthesis, including lipases as biocatalysts. Lipases offer advantages such as facilitating the purification and separation, when immobilized, of reaction products and eliminating the need for conventional catalysts that can leave residues on the product.^16^

The present study highlights the need for a literature review on using lipases in synthesizing aromatic esters. This review will analyze publication patterns, including common keywords, author and research institution collaborations, countries where these studies were conducted, and advances in the field over time, and identify studies that addressed knowledge gaps and explored new research avenues.^17^

By analyzing works on the application of lipases for the synthesis of high-value compounds, we can see research is advancing in several knowledge fields, including chemistry, biology, physics, biotechnology, chemical and materials engineering, and pharmaceutical sciences.^18^

This review aims to provide perspectives for further research by identifying and filling existing knowledge gaps. It also seeks to answer the following questions:

Q1. How has scientific research on the synthesis of esterified flavors using lipases evolved?

Q2. What are the critical points in articles on synthesizing esterified flavors using lipases?

Q3. What are the main perspectives on the topic?

## 2 Methodology

### 2.1 Data Source

A search was conducted using the previously mentioned literature as the basis for the studies.^19–24^ Figure 1 illustrates the methodology used for the analysis. Web of Science served as the reference database, collecting many relevant papers. Articles and bibliographic reviews published between 2014 and 2024 were filtered using the keywords flavor, enzyme, and lipase, resulting in 189 works selected for analysis. The keywords were chosen to show the most significant possible number of works in the area, and filters were applied to choose the best works.

**Figure 1:**
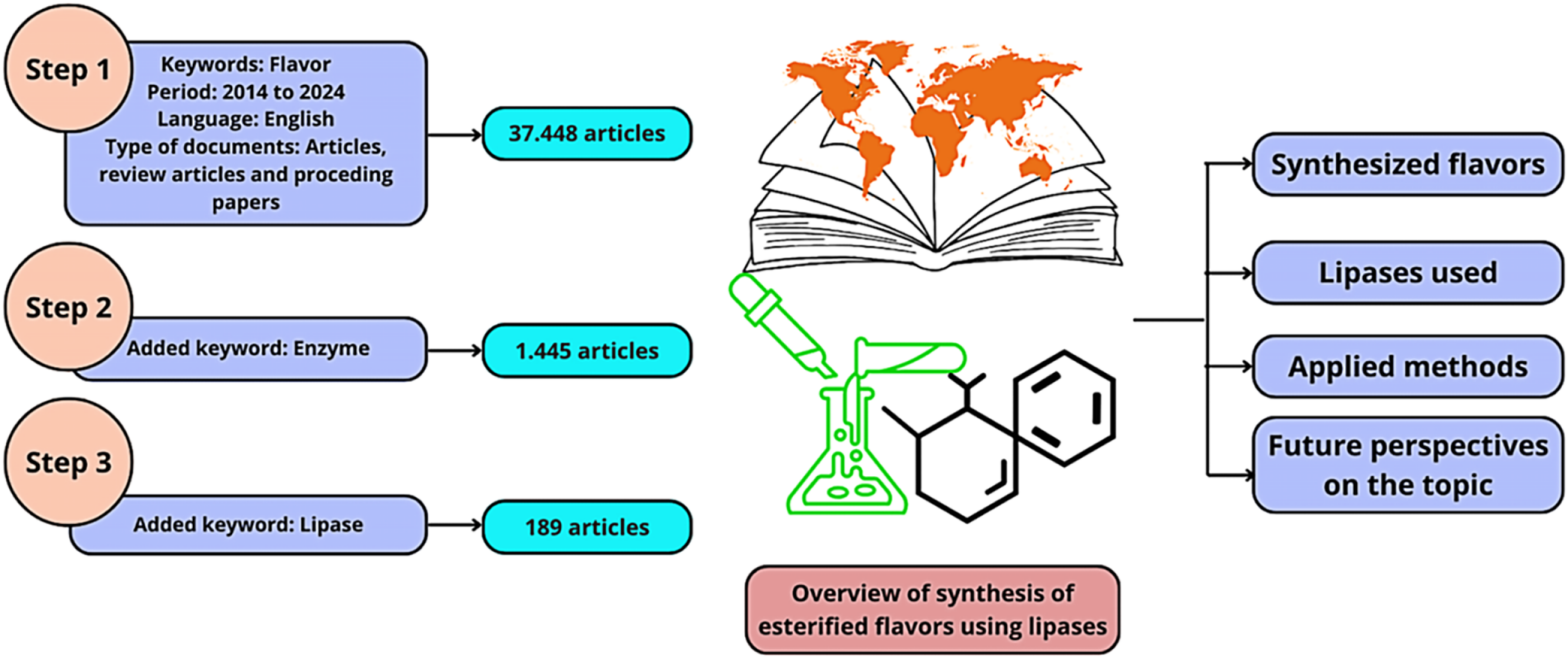
Workflow of the review analysis.

## 3 Results and Discussion

### 3.1 Mechanism of Action of Lipases

Lipases (EC 3.1.1.3) are a subclass of esterase hydrolase enzymes that cleave ester bonds in triglycerides, breaking down fats into glycerol and fatty acids. In most living organisms, lipases play a role in dietary lipids’ digestion, transport, and intracellular processing. To fully understand their function, a thorough review of the mechanism of action of lipases is being conducted, including studies of their structural biochemistry, catalytic operating mechanisms, and specific regulatory mechanisms.^25^

Most lipases comprise a single polypeptide chain that folds into a compact globular structure. Their active site contains a serine, histidine, and aspartic (glutamic) acid triad.^26^ These residues are central to the enzyme’s catalytic mechanism. Another typical feature of lipases is a “lid” domain that covers the active site and mediates conformational changes upon ligand binding.^27^ Figure 2 shows two possible conformations of lipases: the open form, where the lid is open and ligand–active site interaction is favorable, and the closed form, where the lid prevents ligand access to the active site.^28^ The position of this lid controls access to the active site. It is essential for the interfacial activation of the enzyme, a unique feature of lipases that increases enzyme activity in the presence of lipid–water interfaces.^29^

**Figure 2:**
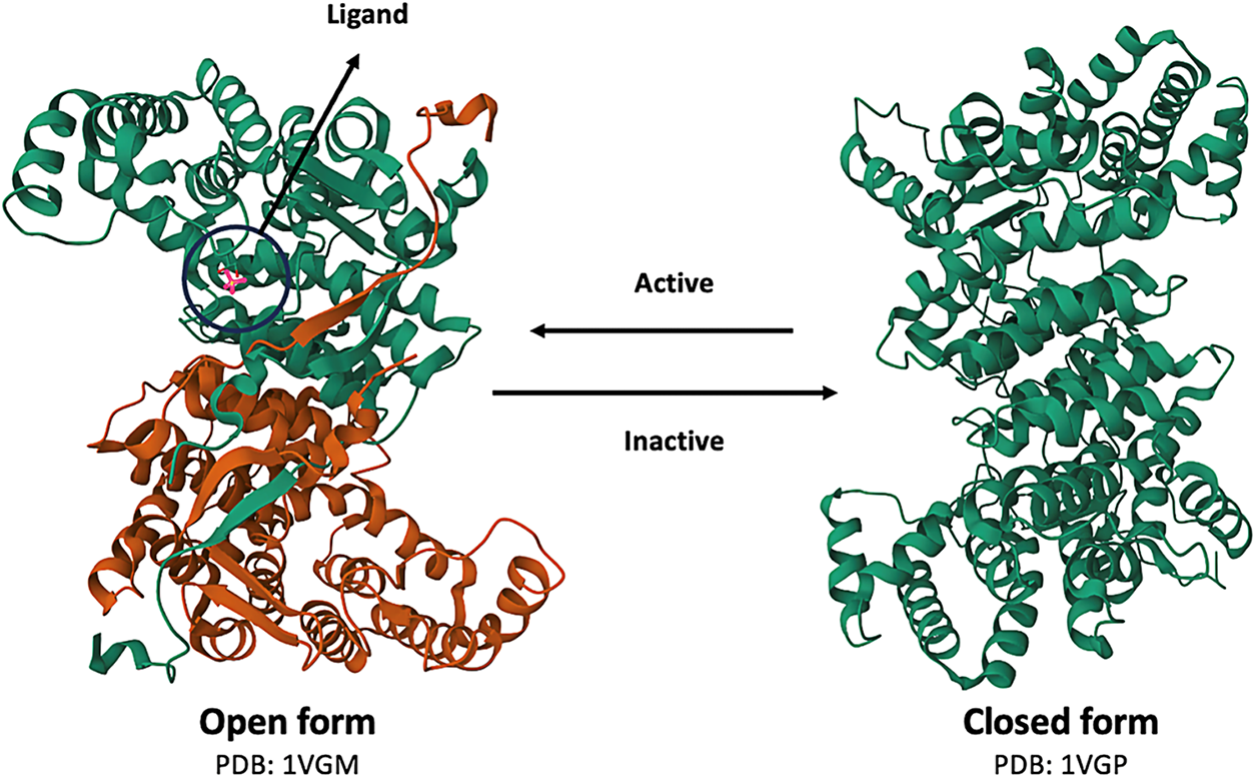
Open and closed forms of lipase, with emphasis on the active site and the lid.

#### 3.1.1 Steps of Esterification and Transesterification Reactions

Lipases operate with complex mechanisms that require specific kinetic and thermodynamic conditions. The lid domain can open to allow the active lipase’s active site to access the lipid droplet substrate.^30,31^ This phenomenon, interfacial activation, is critical to the enzyme’s function. When the substrate, typically a triglyceride, binds to the active site,^32^ an ester bond of the glycerol backbone aligns with the catalytic triad. The serine residue, acting as a nucleophile, attacks the carbonyl carbon of the ester linkage to form a tetrahedral intermediate.^33^ This intermediate is stabilized by an oxyanion hole that accepts the negative charge. The histidine residue, acting as a general base, transfers a proton to the leaving alcohol group, causing the tetrahedral intermediate to collapse.^34^ This results in an acyl-enzyme intermediate and the release of a free fatty acid. Water, activated by the histidine residue, attacks the acyl-enzyme intermediate, forming a second tetrahedral intermediate. This intermediate collapses, releasing the glycerol backbone and regenerating the free enzyme.^35,36^ Figure 3 shows how the mechanism occurs.

**Figure 3:**
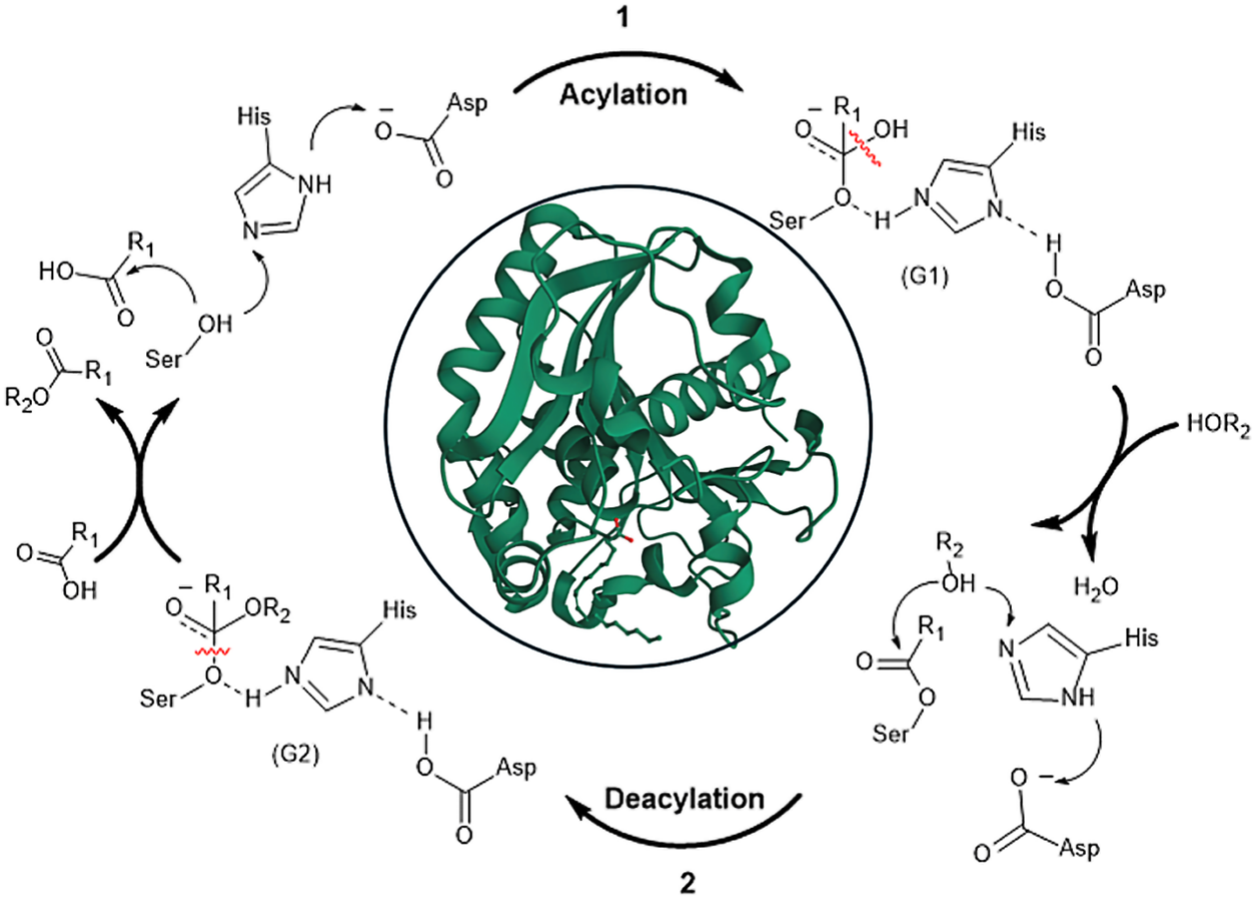
Mechanism of acylation and deacylation of lipases.

#### 3.1.2 Specificity and Selectivity of Lipases

The specificity of lipases is strongly influenced by the geometry and chemical environment of their active sites and binding pockets. These pockets are designed to recognize and bind specific lipid substrates.^37^ Variations in the amino acid residues lining the binding pockets result in differences in substrate affinity and selectivity between different lipases. Lipases exhibit other types of specificity and selectivity, such as regioselectivity, enantioselectivity, and substrate specificity. Substrate specificity refers to the enzyme’s preference for specific lipid molecules. Lipases can be broadly or narrowly specific, operating on various triglycerides or targeting specific fatty acid chains or positions on the glycerol backbone. The shape and composition of the binding sites 28 determine selectivity. Regioselectivity is the preference of lipases to catalyze reactions at specific sites on a substrate molecule. For example, many lipases are more active in cleaving ester bonds at the sn-1 and sn-3 positions of triglycerides than at the sn-2 position.^38^ This specificity results from the spatial arrangement of the active site and the substrate binding. Enantioselectivity refers to the ability of lipases to distinguish between enantiomers (chiral molecules that are mirror images of each other) and to catalyze reactions preferentially involving one enantiomer over the other. This property is beneficial in producing optically pure compounds for the pharmaceutical industry. The chiral nature of the active site allows lipases to select specific enantiomers, resulting in enantioselectivity.^39^

While specificity and selectivity are critical for lipases, they have broader implications for industrial applications and biological systems. In biological systems, lipases are naturally optimized for the efficient digestion and metabolism of dietary fats. In industrial applications, the selective properties of lipases have been widely exploited. For example, in biodiesel production, lipases catalyze the transesterification of methanol with triglycerides to produce biodiesel. In the food industry, lipases modify fats and oils to produce structured lipids with desirable properties. In the pharmaceutical industry, enantioselective lipases are used to synthesize chiral intermediates for drug production.^40^

Lipase isolated from the Candida species exhibited its maximum enzymatic activity at pH 4 and maintained 80 and 90% of its activity at pH 3.5 and 4.5, respectively, indicating sensitivity in an acidic environment; however, at pH 2.0–3.0, the catalytic activity was reduced to 15%–40%.^41^ Vegetable lipases, on the other hand, exhibit more moderate enzymatic activity than microbial lipases and are more specific for their natural substrates. For example, castor bean lipase ( L.) effectively hydrolyzes vegetable oils.^42–44^ In a study by Machado et al., castor bean lipase showed greater specificity for vegetable oils rich in polyunsaturated fatty acids, with oils extracted from the pulp and seeds of macaw and microalgae such as , , and *Chlorella* used in the hydrolytic reaction.^45^ Animal lipases, such as bovine pancreatic lipase, exhibit significant enzymatic activity under physiological conditions (e.g., neutral pH) and are highly efficient in the digestion of triglycerides.^46^ Pancreatic lipases have been reported to effectively catalyze the hydrolysis of primary alcohol esters into valuable products.^47^ However, animal lipases have been the subject of less research than other lipase sources.

### 3.2 Sources of Lipases

Lipases, also known as triacylglycerol hydrolases (EC 3.1.1.3), are enzymes capable of catalyzing various chemical and biochemical reactions and are found in many living organisms.^48^ These enzymes act as biocatalysts in many industrial processes, particularly in the synthesis of aroma esters. The applications of aroma esters cover many sectors, including the cosmetics, pharmaceutical, textile, and food industries, etc.^49,50^ Lipases can be obtained from various sources, including microbial, plant, and animal sources, each with unique characteristics in terms of activity, stability, and substrate specificity.^51–53^ Below is a brief description of different sources of lipase.

Figure 4 shows a three-dimensional structure of the B type lipase, CALB, where one can notice the α-helix, which is called the lid, known for being an amphipathic structure when it is in its closed conformation and the hydrophobic side is facing the catalytic pocket, causing the conformation of the enzyme to contribute to its activity, causing the hydrophobic side to be more exposed or not.^54,55^ Catalysis occurs through substrate and acyl donor interaction with the lipase catalytic triad, which consists of serine, aspartic acid, and histidine, Figure 4.^56^

**Figure 4:**
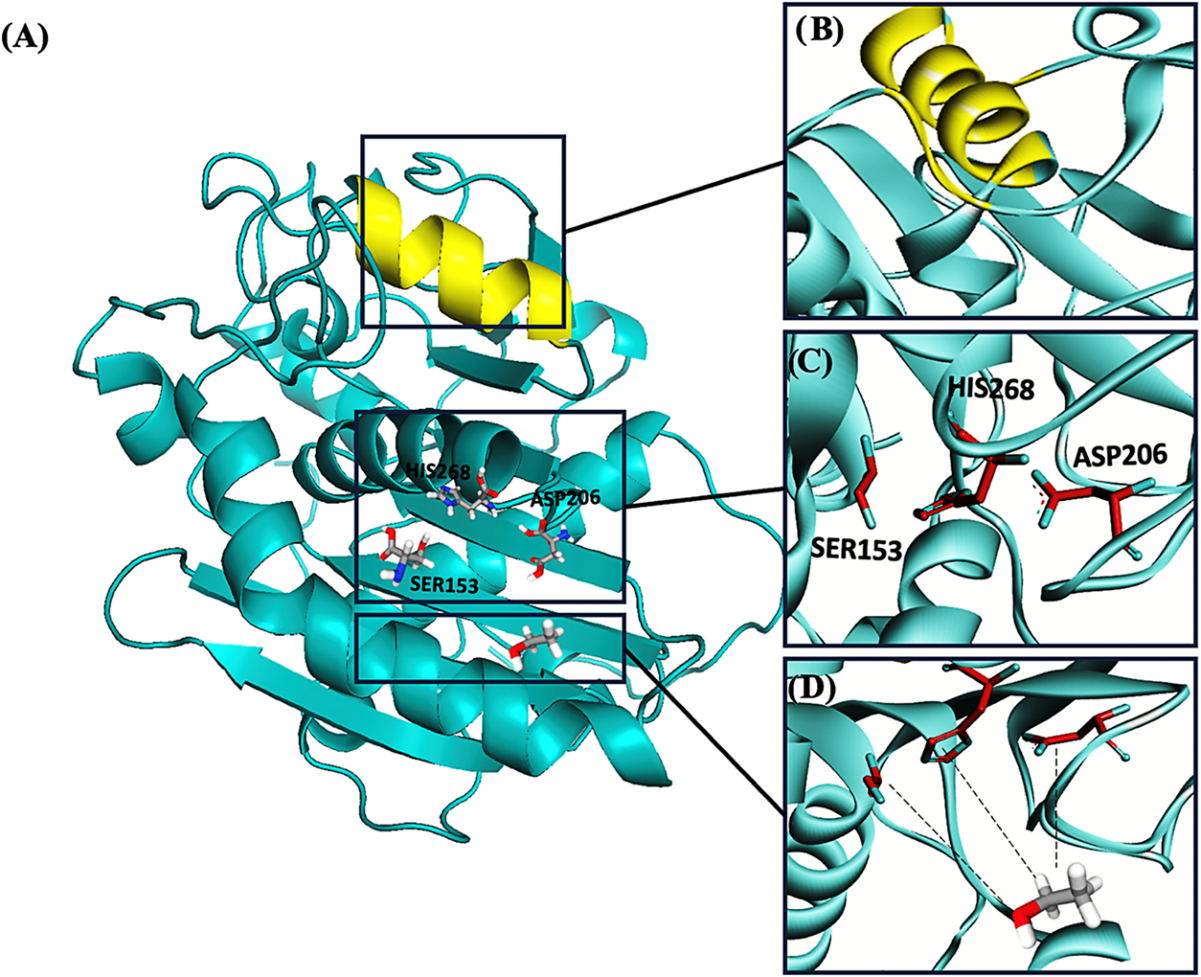
(A) Three-dimensional structure of CALB lipase from (PDB code 1TCA). (B) Catalytic cavity lid present in hydrolase-type enzymes, lid demarcated by residues in the structure (lid 237–267 in yellow). (C) The monomer consists of 317 amino acids. The active site consists of a catalytic triad formed by Ser105, His224, and Asp187. (D) Representation of the intermolecular interaction between the enzyme’s catalytic site and a generic ligand (ethyl alcohol), showing the potential interactions of alcohols and other substrates with CALB lipase.

#### 3.2.1 Diversity of Lipase Sources

Plant lipases are found in various parts of plants, including fruits, leaves, roots, and especially seeds.^57^ A wide variety of seeds such as castor, sunflower, almond, wheat, jatropha, cumin, hazelnut, oat, rice, barley, corn, sorghum, rapeseed, coconut, and sesame can be used to extract these enzymes.^58^ Seeds have higher lipase activity than other parts of the plant due to the high concentration of triacylglycerols, which serve as an energy source for plant growth during germination.^59^ During germination, lipases work with other enzymes to catalyze the breakdown of oils and fats in oilseeds to produce sugars and provide energy and nutrients for the plant.^60^ The recovery of plant lipases can also be an attractive alternative to using agricultural residues, offering lower production costs. They can be extracted from byproducts of fruit processing such as mango, orange, palm, and papaya.^61,62^

Animal lipases are mainly extracted from the pancreas of pigs, cattle, and sheep, although they can also be isolated from the intestinal wall, stomach, and liver of these animals.^59,63^ Extraction of these enzymes may be from specific animal cells or microorganisms present in its gastrointestinal tract.^59^ Besides mammals, lipases can also be extracted from fish and insects, with their primary function being the digestion of lipids and fats.^47^ However, obtaining animal lipases is challenging unless they are produced by genetically modified microorganisms, limiting their commercial application.^64^

Finally, lipases of microbial origin are the most widely used and studied in scientific research and industry.^65^ These lipases are produced by microorganisms such as fungi, bacteria, and yeast.^66^ Among these microorganisms, fungi are considered one of the best sources of lipase due to their extracellular nature, which facilitates extraction and purification for commercial purposes.^67^ This reduces the costs associated with the process, making them a preferred microbial source over bacteria.^67^ Microorganisms that produce lipases can be found in various environments, such as glaciers, hot springs, and contaminated soils.^68^ The most commonly used microbial lipases in biotechnological applications include those from *Candida*, mainly and .^59^ Other widely used microorganisms for this purpose are , , , , and , which are easily obtained by fermentative processes.^69,70^ The microbial lipase market is on the rise. It stands out as one of the most expansive sectors of the lipase industry, as most enzymes currently used in various industrial processes are derived from microorganisms.^71^ In 2019, the global lipase market reached a value of USD 349.8 million and is expected to maintain an annual growth rate of 5.2% during the forecast period from 2020 to 2025, reaching USD 428.6 million by 2025.^68,72^

#### 3.2.2 Comparison of Lipases from Different Sources in Terms of Activity and Stability in the Synthesis of Flavor Esters

Microbial lipases are known for their remarkable catalytic activity and versatility under different reaction conditions.^63,73^ Due to their greater tolerance to pH variations, thermostability, and stability in organic solvents, these enzymes have been increasingly used in hydrolysis and ester synthesis reactions.^70,74^ For example, studies have shown that microbial lipases, especially fungal lipases, are generally stable and active over a wide pH range. Bandi et al. examined the enzymatic activity of free lipase from in solutions with different pH levels, ranging from acidic to primary (pH 4–9).^75^ Researchers found that the maximum lipase activity occurred in a primary environment at pH 8, demonstrating the sensitivity of the lipase to alkalinity.^75^

#### 3.2.3 Limitations of Industrial Applications

The application of lipases in flavor synthesis may have some disadvantages. Regarding the technical aspect, there is a problem with enzyme stability, since lipases are highly quickly inactivated under industrial conditions, such as high temperatures and pH in organic media. However, stability can come through immobilization of the enzyme, but this must occur under strictly controlled conditions, which may be problematic for industrial replication. Another disadvantage is the specificity of the lipase since, in complex systems, it may produce byproducts from the catalysis that occurs.

### 3.3 Case Studies on Flavor Ester Synthesis Using Lipases

Table 1 shows the methodologies of works published between 2018 and 2024, where we can analyze which lipases, types of alcohols, and acyl donors were most used and what the time and temperature averages are, and compare reaction yields.

**Table 1:** Methodologies of Works Published from 2018 to 2024

lipase source	lipase name	alcohol	acyl donor	acyl donor/alcohol ratio	lipase form	time (h)	temp (°C)	yield (%)	ref
*Candida* sp	CSL	methanol	cinnamic acid	1:3 (*v:v*)	purified and immobilized	2	70	90.4	^76^
		ethanol						85.2	
		butanol						98.9	
		geraniol						98.1	
		isoamyl						94.9	
		benzyl						97.3	
*B*	Novozym 435	isoamyl	acetic acid	1:1 (*n:n*)	purified and immobilized	48	60	68	^77^
		pentanol	valeric acid					89.2	
	eversa transform 2.0	methanol	octanoic acid	1:1 (*v:v*)	soluble and immobilized	1.5	25	62.5	^78^
		propanol				1		77.5	
		fusel oil				1.5		65	
		isoamyl				1		82.3	
*B*	CALB-type SRL	ethanol	hexanoic acid	1:1 (*n:n*)	immobilized	20	45	95.1	^79^
	F0215	ethanol	caprylic acid	1:1.2 (*n:n*)	purified	12	40	24.7	^80^
	PPL@mDE	isoamyl	acetic acid	1:1 (*n:n*)	purified and immobilized	3	37	100	^81^
	RML	methanol	butyric acid	1:1.5 (*n:n*)	purified and immobilized	6	25	89 ± 1%	^82^
		ethanol		1:1 (*n:n*)				92 ± 1	
, type VII	MCH@PDA-lipase	isoamyl	acetic acid	1:2 (*n:n*)	purified and immobilized	24	35	98.4 ± 1.3	^83^
		isopentyl						71.5 ± 0.8	
	black cumin lipase	geraniol	butyric acid	1:1 (*n:n*)	microbial lipase	48	37	96	^84^
		citronellol							
Antarctic *Pseudomonas*	AMS8 Lipase	ethanol	hexanoic acid	1:1 (*n:n*)	immobilized	2	20	70	^85^
*B*	CALB	isoamyl	hexanoic acid	1:1 (*n:n*)	immobilized	24	70	93	^86^
			decanoic acid					87	
*B*	CALB10000	isobutyl	propionic acid	1:3 (*n:n*)	immobilized	3	60	95.12	^87^
	Novozym 435	cinnamyl	propionic acid	2:3 (*n:n*)	immobilized	4	50	99.3	^88^
			acetic acid					99	
			butyric acid					96.1	
			valeric acid					98.9	
			hexanoic acid					98.5	
			heptanoic acid					98.5	
			octanoic acid					98.6	
		benzyl	acetic acid					99.5	
			propionic acid					99.8	
			butyric acid					99.6	
		anisyl	acetic acid					99	
			propionic acid					99.1	
			butyric acid					98.9	
	lipozyme TL-IM	geraniol	oleic acid	1:1 (*n:n*)	immobilized	4	35	93	^14^
			lauric acid					91.2	
			stearic acid					82.7	
		citronellol	oleic acid					89.9	
			stearic acid					96.5	
	Novozym 435	geraniol	oleic acid	1:1 (*n:n*)	immobilized	4	35	94.8	
			lauric acid					94.9	
			stearic acid					96.3	
		citronellol	oleic acid					93.9	
			stearic acid					97.6	
lipase	CRL	geraniol	butyric acid	1:1.5 (*n:n*)	immobilized		40	91	^7^
	eversa transform 2.0	geraniol	butyric acid	1:5 (*n:n*)	soluble	6	50	93	^15^
	CRL	isoamyl	acetic acid	1:2.6 (*n:n*)	immobilized	8	50	85.19	^89^
	RSF-PFL	citronellol	vinyl acetate	1:0.25 (*n:n*)	immobilized	12	37	99.8	^74^
	AbEPS	butanol	caprylic acid	1:1 (*n:n*)	immobilized	24	40	95.2	^90^
	LsPS		oleic acid	2:1 (*n:n*)				91.2	
	CPc-PCL	hexanol	vinyl acetate	1:1 (*v:v*)	immobilized	8	37	99	^91^
	CCL on HPMC/PVA	propyl	vinyl benzoate	3:1 (*n:n*)	immobilized	24	55	99	^92^
Porcine pancreas	CA-CS-CNTs-PPL	ethanol	hexolic acid	1.1:1 (*n:n*)	immobilized	20	40	88	^93^
lipase B	Novozym 435	octanol	formic acid	1:7 (*n:n*)	immobilized	1	40	80.71	^94^
lipase	EO-proROL	isoamyl	butyric acid	1:1 (*n:n*)	immobilized	5	30	84	^95^
			acetic acid	1:8 (*n:n*)		24		84	
Porcine pancreas	PPL-ILs/Fe3O4@MO	isoamyl	acetic acid	1:1 (*n:n*)	immobilized	12	60	75.1	^96^
lipase B	Novozym 435	pentanol	butyric acid	2:1 (*n:n*)	immobilized	4	30	99.8 ± 0.2	^97^
		octanol						99.3 ± 0.1	
		dodecanol						99.6 ± 0.2	
		pentanol	hexanoic acid					99.9 ± 0	
		octanol						98.9 ± 0.1	
		dodecanol						99.5 ± 0.6	
lipase B	Novozym 435	ethanol	valeric acid	1:0.5 (*n:n*)	immobilized	0.66	50	69.2	^98^
lipase B	Novozym 435	benzyl	butyric acid	1:1 (*n:n*)	immobilized	24	50	80	^99^
Porcine pancreas type II	CCAC-PPL	isoamyl	acetic acid	1:1 (*n:n*)	immobilized	4	40	93.23 ± 0.12	^100^
	CCACM-PPL							96.62 ± 0.85	
strain	An605, An1097, and An3131	ethanol	caprylic acid		whole cell	8	28	94.80	^101^
			capric acid					85.20	
lipase	CRL-Diaion HP 20	hexanol	butyric acid	1:1 (*n:n*)	immobilized	1	60	99.5	^102^
lipase B	Novozym 435	butanol	caprylic acid	1:2 (*n:n*)	immobilized	3.5	60	95.33	^103^
lipase B	Novozym 435	pyridine methanols	ethyl carboxylates	5:1 (*n:n*)	immobilized	36	50		^104^
lipase B	CALB	geraniol	acetic acid	1:1 (*n:n*)	immobilized	0.16	50	70	^105^
lipase type VII	CRL/SiO_2_/Fe_3_O_4_/GO	ethanol	valeric acid	1:2 (*n:n*)	immobilized	3	40	90.4	^106^
fungus	lipozyme RM-IM	benzyl	benzoic anhydride	1:5 (*n:n*)	immobilized	24	40	51	^107^
	lipozyme	pentanol	acetic acid	1:1 (*n:n*)	immobilized	8	40	80	^108^
	SNPs-Est	ethanol	pyruvic acid	1:1 (*n:n*)	immobilized	11.3	45	96	^109^
	MNC/PES-AOL	ethanol	valeric acid	1:1 (*n:n*)	immobilized	24	50	72	^110^
	NC-SiO_2_-PES/CRL	pentanol	valeric acid	1:2 (*n:n*)	immobilized	6	50	60.4	^111^
lipase B	Novozym 435	isoamyl	acetic anhydride	1:1 (*n:n*)	immobilized	2	50	100	^112^
Steapsin lipase	Sc-CO_2_	benzyl	butanoyl donor	1:2 (*n:n*)	immobilized	7	48	99	^113^
	RL/SiO_2_/Fe_3_O_4_/GO	ethanol	valeric acid	1:1 (*n:n*)	immobilized	16	30	77	^114^
	CRL-Diaion HP 20	hexanol	butyric acid	1:2 (*n:n*)	immobilized	8	47	95	^102^

When analyzing the donor species, we can see an emphasis on , as this lipase is highly versatile when it comes to being used as a biocatalyst. However, emphasis must be placed on the most used lipases, CALB and Novozym 435, which are lipases derived from , all of which are used in immobilized form due to the low loss of activity if it is necessary to increase the reaction temperature and carry out reuse to analyze enzymatic efficiency. Most articles analyzed how immobilized lipase behaves in the esterification reaction, comparing the free enzyme with the immobilized one.

Another observation is that alcohols and acyl donors with small chains showed better yields, as they have lower steric hindrances when they come into contact with biocatalysts, which may justify the alcohol:acyl donor ratio being 1:1 on most jobs. As most of these reactions do not have any steric hindrance due to the size of their molecules, catalysis can occur quickly and precisely at mild temperatures to maintain the stability of the biocatalyst.

Another factor to note is that in most studies, the lipases used are immobilized, offering more excellent stability to catalyze reactions at higher temperatures or allowing reuse cycles. The authors compared immobilized and free-form enzymes to produce a well-elaborated study. In all of the studies where this comparison was made, the results favored the immobilized lipases, showing higher conversion rates.

Ethanol and isoamyl alcohol were the alcohols that showed the most efficiency in the reactions, which can be explained by their small structural size, facilitating the chemical reaction and reducing the steric effect caused by larger molecules in the reaction medium. On the other hand, acetic acid was the most commonly used acyl donor, which can be justified by the same reason as the alcohols and its mild acidity.

An essential factor in a chemoenzymatic reaction is time. By analyzing the studies, we can see that in most cases, the reactions occur very quickly, and in just a few hours, a product with high conversion can be obtained, even at room temperature.

However, in other studies, we can see that there is a long period to obtain the products, in addition to high temperatures, which can cause denaturation of the lipase; therefore, for these studies, it is recommended to work more on the reaction conditions, both time and temperature.

### 3.4 Optimized Reaction Conditions

#### 3.4.1 Study the Influence of Temperature, pH, Substrate, and Solvent Concentration on Reaction Performance

The synthesis of aromatic esters using lipases is an enzymatic process influenced by many factors. These factors can be grouped into enzyme selection, reaction conditions, and reaction system components.^115^ Lipases have great potential to significantly drive the growth of the bioprocessing industry due to their versatile applications in catalyzing a wide range of reactions, especially in the bioprocessing of raw materials and the synthesis of organic chemicals.^116,117^ Several factors contribute to the application of lipases, including their regiospecific, enantiospecific, and chemospecific nature, their ability to catalyze reactions in both aqueous and nonaqueous media, and their ability to catalyze both direct and indirect reactions.^116,118^ In their immobilized form, lipases offer several processing advantages, such as easy separation from the product mixture, improved stability, and the ability to operate continuously, all of which are essential for any production process.^116^

Using immobilized enzymes to synthesize various products aims to achieve high process specificity, selectivity, productivity, and easy recovery. Several factors can influence the activities of these biocatalysts, including the enzyme source and the nature of the immobilization support.^119^ One of the essential properties of immobilized enzymes is the possibility of their recovery and reuse, which are significant economic and environmental aspects that can determine their future industrial applications.^120,121^ Another critical factor is the amount of enzymes that deserve economic and industrial viability attention. The amount of enzyme can significantly affect the whole process. A more significant amount of biocatalyst will dramatically contribute to forming the enzyme–substrate complex, resulting in a higher conversion. However, an excessive amount of enzyme can lead to clumping, which blocks substrate sites from enzyme attack. Therefore, excess enzymes may not contribute to the reaction rate and must be controlled to ensure high conversions and low costs.^122,123^

Studying the molar ratio of substrates is significant, as it determines the influence of substrates on enzymatic activity and mass transfer in the reaction system. High molar ratios facilitate the rapid formation of the enzymatic acyl complex, while low molar ratios may limit mass transfer and slow the process. When there is an excess of a substrate, the reaction equilibrium shifts toward product formation.^124^ However, high acid concentrations can inhibit catalytic activity and reduce enzyme efficiency.^122^ To counteract the inhibitory effect of acid during esterification, some researchers recommend using a higher alcohol concentration. However, increasing the amount of alcohol too much can also decrease the conversion. This is due to the polar nature of alcohol, which interacts hydrophilically with the water layer on the surface of the enzyme, causing changes in the protein structure of the enzyme, leading to inhibition and reduced activity.^48,125,126^

The presence of water can affect the stability of the enzyme in several ways. For example, it affects the enzyme’s structure by forming and breaking noncovalent hydrogen bonds, facilitates the diffusion of reagents, and involves the reaction equilibrium. Both very low and very high water contents can affect enzyme activity. Low water content can reduce enzyme activity, while high water content can reduce reaction rates by aggregating enzyme particles and limiting diffusion.^127,128^ Esterification is generally limited in water, because the equilibrium catalyzed by hydrolytic enzymes tends to favor hydrolysis (the reverse reaction). Since esterification produces water as a byproduct, its removal by molecular sieves can improve ester synthesis by shifting the reaction equilibrium forward.^48^

Analysis of the need for and effect of solvents in the reaction media is critical to the viability of the process. Certain solvents can aid solubilization and optimization of conditions for product conversion, but they can also add cost to the process.^129^ Solvents used in enzymatic esterification can mitigate these problems in the reaction medium. Solvents play an essential role in the solubility of substrates and can significantly affect reaction yield when substrates have low mutual solubility.^130^ However, the use of solvents also introduces additional costs and environmental concerns. To overcome these drawbacks, using solvent-free systems for enzymatic esterification has increased interest in recent decades.^131,132^ The nature of the solvent influences the activity, selectivity, and stability of the enzymes. Lipases are generally more stable when suspended in nonpolar solvents with low water solubility. The choice of organic solvent for enzymatic reactions is essential to provide good substrate solubility in the reaction medium without compromising the enzyme’s catalytic power.^48,122^

Temperature is a critical parameter in biocatalysis because it facilitates the solubility of reactants in reaction media, reduces the viscosity of the mixture, increases the molecular collision interface, reduces mass transfer limitations, and facilitates interactions between enzyme particles and substrates.^125,126,133^ Temperature has a direct effect on the lipase activity and reaction rate. Higher temperatures can increase the reaction rate and activate the enzyme. Therefore, finding an optimal temperature that maximizes enzyme activity without causing denaturation^39,114,125^ is critical.

In mild environments, ester synthesis can be performed by enzymes, such as lipases. To maintain enzymatic activity, the pH of the medium should be as appropriate as possible. Each enzyme has an ideal pH for the highest catalytic activity.^134^ The choice of proper pH depends on the type of catalysis and the substrate used. Significant deviations from this perfect pH can deactivate the enzyme and reduce the rate of ester formation. An acidic pH is required for acidic esterification, whereas a basic pH may be more appropriate for transesterification.^134^

To maximize the yield and purity of aroma esters, it is essential to maintain a stable pH during the reaction. Standard techniques for maintaining the desired pH include gradually adding catalysts and controlling the temperature. One of the components that essentially contributes to the reaction rate is pH. Analyzing the medium’s pH is necessary to synthesize the desired ester.^135,136^

#### 3.4.2 Strategies for Optimizing Reaction Conditions

Several strategies can optimize the reaction conditions. These include selecting the most appropriate enzyme for the reaction, determining its application form (free or immobilized), and adjusting the components’ molar ratio and the experiment’s temperature.^137,138^

The study entitled “Comparison of the performance of commercial immobilized lipases in the synthesis of different flavor esters”^121^ compared the performance of three commercial lipase preparations (Novozym 435, Lipozyme TL-IM, and Lipozyme RM-IM) in the synthesis of flavor esters. Esterification of acetic, propionic, and butyric acids with various alcohols (ethanol, isopropanol, butanol, and pentanol) was performed.^121^ The results showed that Novozym 435 was the most efficient and suitable enzyme preparation for the synthesis of aroma esters, outperforming Lipozyme TL-IM and Lipozyme RM-IM. In general, reactions with longer chain acids resulted in higher yields. Novozym 435 outperformed in most cases, except for the production of ethyl butyrate, where the Lipozyme RM-IM showed better performance. Reactions with butyric acid gave the highest conversions for all of the biocatalysts. After optimization of the reaction conditions, all three enzymes achieved yields above 90%, although Lipozyme TL-IM required four times more biocatalyst. Regarding reuse, Novozyme 435 retained more than 80% of its activity after nine consecutive cycles, while Lipozyme RM-IM and Lipozyme TL-IM could be reused five and three times, respectively. Therefore, Novozym 435 proved to be the most efficient enzyme, requiring less biocatalyst and offering more excellent durability in reuse.^121^

Another study entitled “Enzymatic Synthesis Of Geranyl Butyrate Via Esterification Catalyzed By Lipase Immobilized On A Hydrophobic Support”^139^ discusses the preparation of a biocatalyst by immobilizing lipase on hydrophobic polystyrene-divinylbenzene particles, which was used in the synthesis of geranyl butyrate via the esterification of geraniol and butyric acid in hexane. The biocatalyst exhibited a high concentration of immobilized protein (39 mg/g of support) and a hydrolytic activity of 437.4 ± 23.6 U/g^–1^. The conditions that maximized ester synthesis were a biocatalyst concentration of 10% m/v, a temperature of 40 °C, and a concentration of 1 mol/L of each reagent, achieving 85% conversion in 3 h. The biocatalyst retained 65% of its initial activity after five reaction cycles, indicating its potential for aroma ester synthesis by esterification.^139^ The study concludes that the prepared biocatalyst could be a significant alternative in synthesizing industrially important aroma compounds.^139^

Another related study, “Enzymatic Synthesis of Isopentyl Caprylate Using Fusel Oil as Feedstock,”^140^ focuses on the optimization of reaction parameters for the synthesis of a natural flavor from a monoterpenic alcohol (isopentanol) and caprylic acid, catalyzed by Novozyme NZL-102-LYO-HQ (Cal B (PU)). Four commercial lipase preparations covalently immobilized on epoxy-polysiloxane-β-cyclodextrin were tested as potential biocatalysts for the esterification of isopentanol with caprylic acid in a solvent-free medium. immobilized lipase was the most active biocatalyst, achieving an ester conversion of over 80% in 24 h.^140^ An experimental design and analysis revealed that isopentyl caprylate formation was strongly influenced by the variable molar ratio at the 95% confidence level. The proposed mathematical model predicted that excess acrylic acid (fusel oil to an acid molar ratio of 1:1.5) and a reaction temperature of 45 °C favored high ester conversion.^140^

These studies underscore the importance of exploring different approaches to optimize the synthesis of flavor esters and highlight the critical role of biocatalysts in the efficiency and sustainability of industrial processes.^141^ Using immobilized enzymes increases the reaction efficiency and allows the reuse of biocatalysts, making processes more economical and environmentally friendly. Careful selection of reaction parameters maximizes the yield and product quality. Diversification of lipase types and immobilization supports allows customization of synthesis processes to meet specific industrial needs. Different substrates will enable the production of a wide range of esters with different aromatic properties. Therefore, continuous research and development of new optimization strategies are essential to advance the production of aroma esters and meet the growing demand for high-quality products.^142^

### 3.5 Bioreactor Designs for Flavor Esters Synthesis Using Lipases

Developing bioreactors for the enzymatic synthesis of flavor esters is a scientific advance and a potential game-changer for the food industry.^143–145^ The bioproduct in question, widely used as a flavor and aroma agent in various products, could see a significant shift in the production methods. The benefits of biocatalysis (e.g., energy efficiency, specificity, and reduced environmental impact) are practical solutions to real-world challenges.^146–148^

Several bioreactors can be used in the enzymatic synthesis of esters, each with characteristics that affect the process’s quality, effectiveness, and efficiency.^149,150^ Notable types include batch bioreactors, fluidized bed bioreactors, fixed-bed bioreactors, and membrane bioreactors.^151–153^ Table 2 lists the types of reactors commonly used for the biocatalytic synthesis of aroma and flavor esters, along with the practical implications of the advantages and disadvantages of each type.

**Table 2:** Types of Reactors Commonly Used in the Synthesis of Flavor Esters

type of bioreactor	benefits	disadvantages
batch	versatile in facilitating various reactions and providing effortless control of operational parameters	large-scale processes experience downtime due to cycle feeding, resulting in reduced efficiency
continuous flow	reduced downtime for feeding, offering greater productivity and efficiency	strict control of operating parameters and robust configuration
fixed bed	continuous operation and high stability of the immobilized enzyme, resulting in reduced enzyme degradation	resistance to flow caused by clogging problems and difficulty in homogenizing the enzyme–substrate complex
membrane	separation of the enzyme and the product	high operating cost and frequent membrane maintenance

In fixed-bed bioreactors, the contact between the enzyme and the substrate is extensive, resulting in high conversion rates.^154^ This is because the enzymes are immobilized on a solid support through which the substrate is continuously passed.^155^ Immobilization allows continuous enzyme use, increasing their efficiency.^156^ However, efficiency can be limited in large-scale systems due to the concentration gradient along the bed.

The configuration of membrane bioreactors allows for the reuse of enzymes and contraction parameters (e.g., pH and temperature).^157,158^ This is a significant advantage because it reduces the need for frequent enzyme replacement and allows fine-tuning reaction conditions, resulting in more efficient and cost-effective processes.^159^ In addition, membranes separate the biocatalyst, the substrate, and the resulting products.^160^ This configuration also increases stability, preventing inactivation and extending the useful life of the biocatalyst.^161^

#### 3.5.1 Patents Related to Flavor Ester Synthesis Using Lipases

Beyond academic research, industry has also recognized the significant potential of lipases in aroma synthesis. This is evidenced by Figure 5, which presents data on patents for flavor esters by lipases. In patent database searches, 44 patents related to flavor ester synthesis were found over the past 7 years, with none registered in 2024.^162,163^ Figure 5A reveals that 2023 had the highest number of patents, totaling 6 registrations. Figure 5B indicates that China leads with 61% of the patents filed, followed by Japan with 26%.

**Figure 5:**
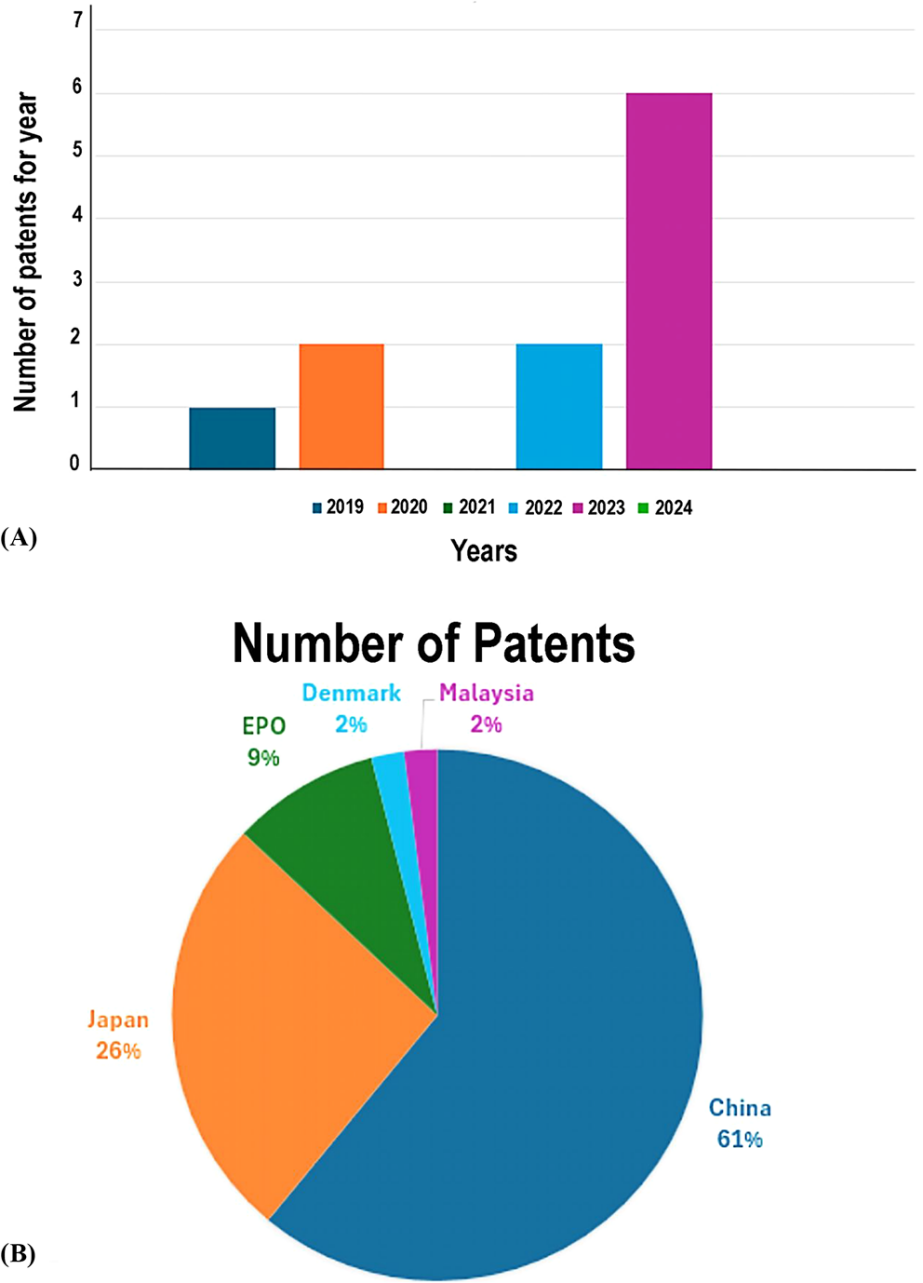
Patent data are related to aroma synthesis by lipases. (a) Evolution of the number of patents over the last 7 years. (b) Countries with the most patents filed.

### 3.6 Perspectives—Futures and Gaps

The use of lipases as biocatalysts in aroma synthesis has a promising future due to their specificity, efficiency, and versatility under mild reaction conditions.^164^ Lipases can catalyze reactions such as esterification, transesterification, and hydrolysis, producing high-purity aromatic compounds.^165^ Advances in enzyme engineering and lipase immobilization increase stability, reusability, and enzymatic activity, making biocatalytic processes more economical and sustainable.^166,167^ Besides, integrating new technologies, such as microwave- and ultrasound-assisted biocatalysis, can further improve reaction efficiency and yield, expanding the industrial applications of lipases in aroma synthesis.^168^

Despite significant progress, challenges remain in maximizing the potential of lipases in aroma synthesis.^169,170^ An important limitation is the need for lipases with high operational stability and tolerance to organic solvents commonly used in aroma synthesis reactions. A solution to this could be the application of natural deep eutectic solvents (NADES), as they present a green alternative to conventional organic solvents^171^ They are exploring and modifying new lipase sources and developing advanced immobilization techniques are essential to overcome this barrier.^172,173^ In addition, a deep understanding of reaction mechanisms and enzyme–substrate interactions is crucial to optimize reaction conditions and minimize unwanted byproducts.^174^ Interdisciplinary collaboration among biologists, chemists, and engineers can accelerate the development of innovative solutions and enable lipase biocatalysis to become a standard tool in the industrial production of flavors.

This paper shows the growing number of publications aimed at synthesizing esterified flavors using lipases. We can see that many studies use various lipases for synthesis, such as , such as CALB, Lipozyme, and Novozym 435. However, many of these lipases are immobilized; thus, enzyme reuse is studied. It is worth noting that the temperatures used in the articles found are between 20 and 70 °C, showing that the lipases act at room temperature, and those that act at higher temperatures are immobilized due to the thermal stability of the support. What is also shown is how the conformation of lipases can be when they are being used as biocatalysts in a hydrolysis reaction, as well as in an acetylation reaction. Another result shown in this work is the growing number of patents on the subject. This work is expected to contribute to the literature and show much to be researched on the subject.
